# Distribution and factors associated with urogenital schistosomiasis in the Tiko Health District, a semi-urban setting, South West Region, Cameroon

**DOI:** 10.1186/s40249-021-00827-2

**Published:** 2021-04-12

**Authors:** Adeline Enjema Green, Judith Kuoh Anchang-Kimbi, Godlove Bunda Wepnje, Vicky Daonyle Ndassi, Helen Kuokuo Kimbi

**Affiliations:** 1 Department of Zoology and Animal Physiology, Faculty of Science, University of Buea, P.O. Box 63, Buea, Cameroon; 2 Department of Medical Laboratory Sciences, Faculty of Health Sciences, The University of Bamenda, P.O. Box 39, Bambili, Cameroon

**Keywords:** Urogenital schistosomiasis, Prevalence, Distribution, Risk factor, Tiko Health District, Cameroon

## Abstract

**Background:**

Increased risk of schistosomiasis in peri-urban and urban towns is not uncommon. An epidemiological survey was carried out in the Tiko Health District (THD), an unmapped transmission focus for urogenital schistosomiasis (UGS), to assess the distribution, intensity, and risk factors associated with the occurrence of UGS.

**Methods:**

In this cross-sectional survey, 12 communities were purposively selected from four health areas (HAs) (Likomba, Holforth, Holforth-Likomba, and Mutengene) in South West Region of Cameroon between June and August 2018. Consenting individuals were enrolled using a convenient sampling technique and administered a semi-structured questionnaire to document information on socio-demographic and water contact behaviour. Urine samples were examined for *Schistosoma* *haematobium* infection using test strip, filtration, and microscopy methods. Bivariate and binary logistic regression analyses were used to identify predictors of infection.

**Results:**

The overall prevalence of UGS in Likomba, Holforth-Likomba and Holforth was 31.5% [95% confidence interval (*CI*): 28.3–34.8] with geometric mean (GM) egg count of 28.7 (range: 2–450) eggs per 10 ml of urine. *S.* *haematobium* infection was not found in Mutengene HA. Infection was unevenly distributed among the HAs, Holforth-Likomba and Holforth being the most and least affected, respectively. The prevalence of infection varied (*P* < 0.001) among the affected communities, ranging from 12.0 to 56.9%. Infection status of the community related positively (*P* < 0.001) with proximity to stream (< 100 m), the degree of contact with water and number of improved water sources. Younger age group (5–14 years) [adjusted odds ratio (*aOR*): 3.7, 95% *CI*: 1.1–12.2] and intense water contact (degree II) (*aOR: *5.2, 95% *CI*: 3.4–8.1) were associated with increased risk of infection. Similarly, significantly higher egg load was observed among younger aged groups (*P* = 0.02) and those who carried out intense water contact activities (*P* < 0.001).

**Conclusions:**

Generally, THD is a moderate risk endemic focus for UGS but prevalence higher than 50.0% was observed in some communities. These findings warrant immediate mass chemotherapy with praziquantel to reduce morbidity. Provision of portable water and health education are proposed measures to reduce and eventually eliminate transmission in the area.

**Graphic abstract:**

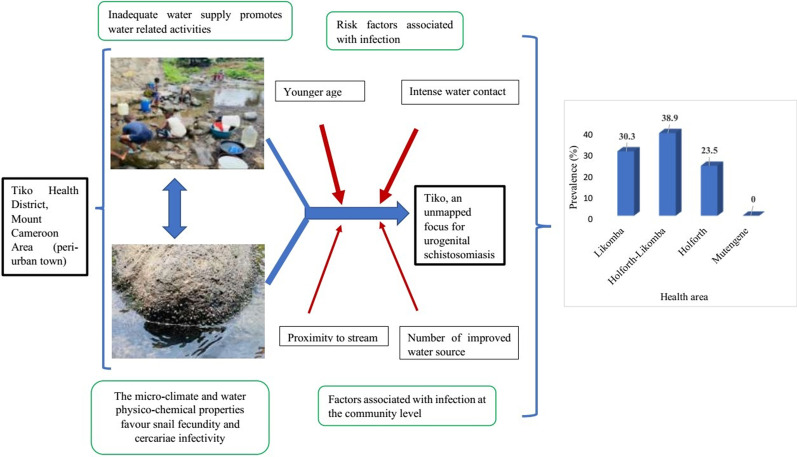

**Supplementary Information:**

The online version contains supplementary material available at 10.1186/s40249-021-00827-2.

## Background

Schistosomiasis is the third highest burden among the neglected tropical diseases (NTDs) and is endemic in 78 countries worldwide, where an estimated 800 million people are at risk of the disease [1]. Of the 250 million infected individuals, 200 million live in Africa where the two predominant disease sub-types are intestinal and urogenital schistosomiasis caused by *Schistosoma* *mansoni* and *S.* *haematobium*, respectively [2, 3]. Globally, an estimated 1.43 million disability-adjusted life years (DALYs) is lost to schistosomiasis [4]. Over 150 000 deaths are attributable to chronic infection with *S.* *haematobium* in Africa [5, 6].

The transmission of schistosomiasis is governed by social–ecological systems such as conditions of poverty and living near open freshwater bodies [7]. Children are at a greater risk of acquiring the infection as well as re-infection [8], and this might lead to growth retardation, anaemia and low school performance [9]. Nonetheless, in endemic areas, where there is lack of adequate water supply, poverty, ignorance and poor hygienic practices, any demographical groups, irrespective of age or gender in contact with unsafe water is at risk of infection [10]. The current mainstay of schistosomiasis control is preventive chemotherapy—the periodic administration of praziquantel to at-risk groups (e.g., school-age children). While this strategy does not prevent infection or reinfection, it reduces morbidity and might also impact on transmission [11]. Schistosomiasis can be prevented by avoiding contact with contaminated freshwater, and the risk of infection can be reduced through improved access to water, sanitation, and hygiene (WASH), information, education, and communication (IEC) [12].

Although schistosomiasis is classically described as a rural disease that occurs in areas without potable water and adequate sanitation [13], urban foci of parasites in tropical areas can no longer be ignored [14]. Increased risk of schistosomiasis in urban areas has been linked to migration of people to neighbouring towns for more attractive employment opportunities [13, 15], water resource development to curb inadequacy in water supply [16] and small multipurpose dams that may lead to ecological changes [17, 18]. Outbreak of neglected tropical diseases in urban areas has been reported due to rapid urbanization which at the same time can also fail to sustain healthy populations when it surpasses clean water reserve and sewage management systems [19]. More so, in this era of global warming and climate change, the epidemiology of temperature dependent infectious diseases is rapidly changing [16].

In the South West Region of Cameroon, records of urogenital schistosomiasis are mostly from rural areas [8, 20–23]. An unmapped transmission focus has been reported in peri-urban communities in the Tiko Health District (THD) [24]. It remains unknown whether urogenital schistosomiasis (UGS) has existed in the THD for an undetermined period or has recently been introduced. In low and middle-income countries experiencing urbanization, the problem of access to water and sanitation is more critical in secondary cities because of the lower basic infrastructure compared with the situation in the primary cities [18]. Inadequate water supply and sanitation has been reported as a major concern in the THD [25] and in addition to extensive rural–urban migration probably due to interurban trading or political unrest in the English-speaking part of Cameroon [26], transmission intensity of UGS may be exacerbated in this peri-urban setting. The previous survey conducted in this new focus was geographically limited to a single community and involved only children aged 5–20 years [24]. In addition, a less sensitive method (urine sedimentation) was used for the diagnosis of *S.* *haematobium* infection and this might have underestimated true infection levels. In view of these limitations and to identify the mechanisms for transmission and maintenance of this disease in THD, an epidemiological survey was carried out among the different population groups, to assess the distribution, intensity as well as describe the risk factors associated with occurrence of UGS in the study area.

## Materials and methods

### Study area

**Fig. 1:**
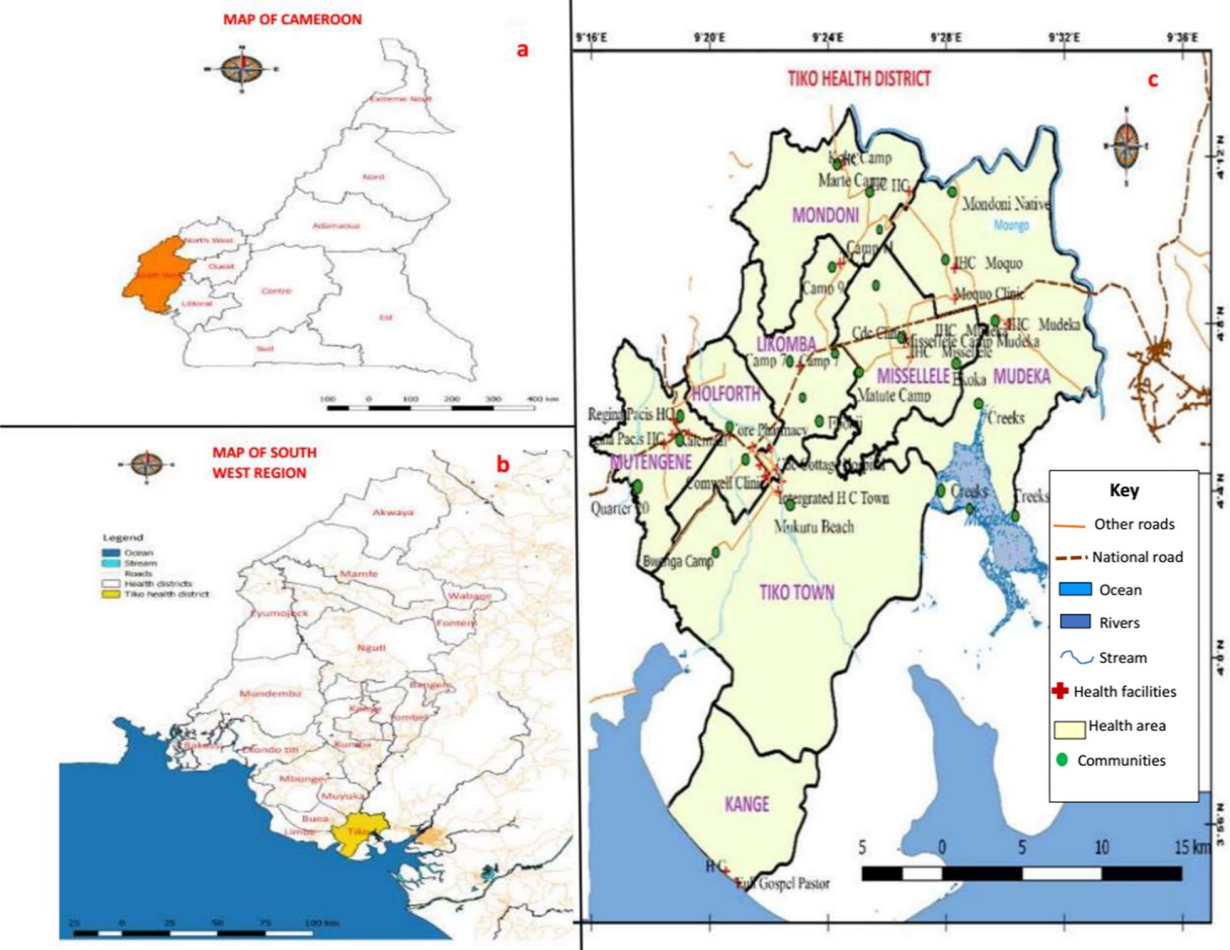
Maps showing location of study site **a** Map of Cameroon showing the location of South West Region. **b** Map of South West Region. **c** Map of Tiko Health District

This study was carried out in the THD, located in Fako Division of the South West Region of Cameroon. The coordinates of THD ranged from altitude 18 m, latitude 9° 32′ 2″ N to 9° 40′ 9″ N to altitude 220 m, longitude 9° 25′ 7″ E to 9° 55′ 7″ E, with a total surface area of 4840 km^2^. This Health District is not geographically isolated from the UGS focus in Munyenge, Bafia Health Area, Muyuka Health District (Fig. 1b). Tiko health district is made up of eight HAs (Holforth, Kange, Likomba, Mutengene, Mondoni, Mudeka, Missellele, and Tiko Town) (Fig. 1c) and inhabited by a heterogenous population of approximately 124 423 inhabitants [27]. This area is characterized by the presence of a local seaport that allows for fishing, import as well as export of goods between neighbouring countries. The rich volcanic soil encourages farming activities and industrial agriculture. This health district hosts the Cameroon Development Corporation (CDC) plantations where banana, rubber and oil palm are cultivated and exported.

**Fig. 2:**
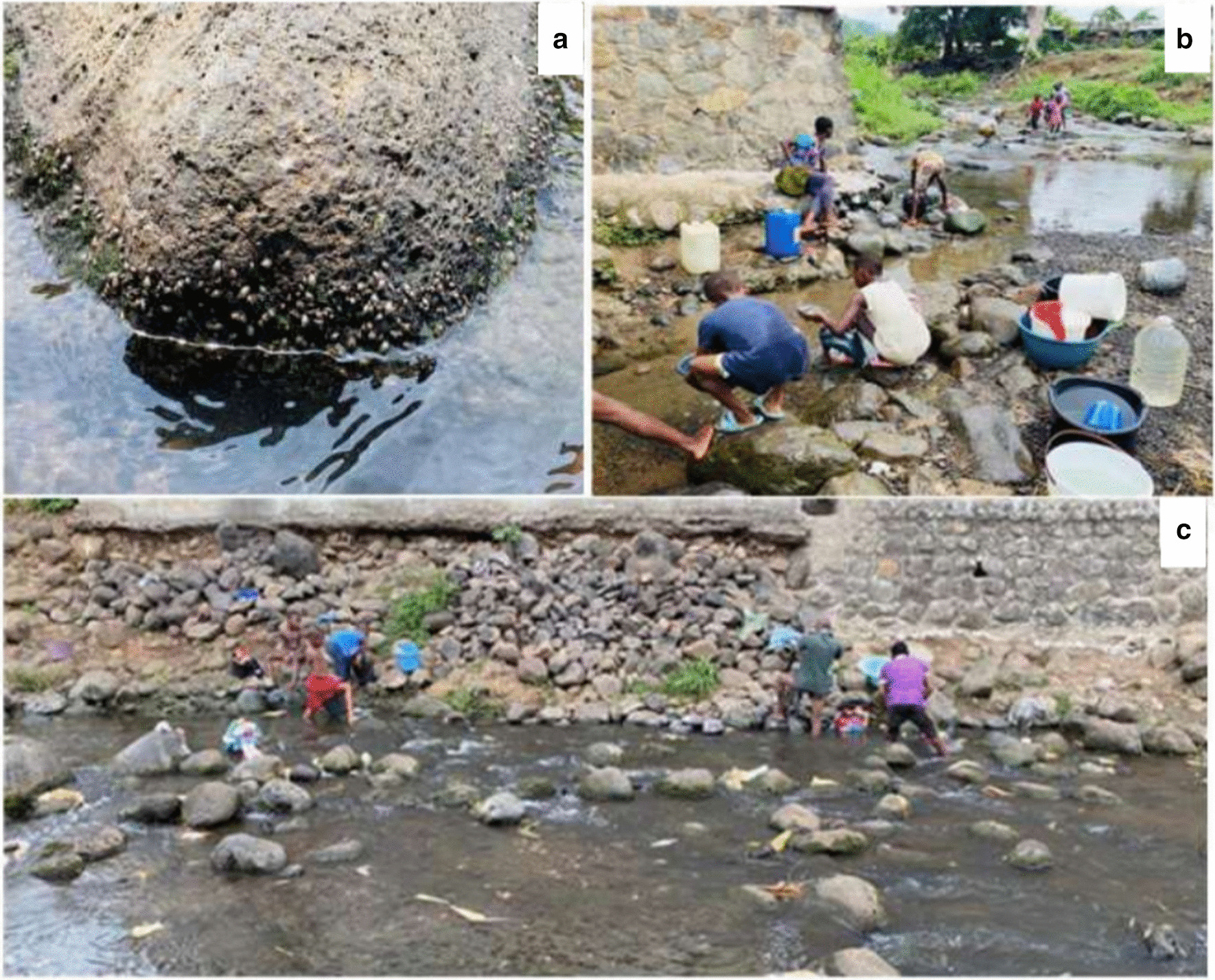
Photographs of snail infested open water and human water contact behaviour in an affected community. **a** Snail infested stream, **b **and** c** Typical stream contact activities

Tiko Health District has a coastal equatorial climate with two distinct seasons: a rainy season, which lasts from March to October and a short dry season of four months (November–February). The mean annual rainfall is about 4524 mm. The temperature is relatively uniform throughout the year, with daily mean temperatures ranging from 28 to 33 °C [25]. This temperature range favours the development rate of the parasite within snails and the infectivity of cercaria [28]. The main water courses in the Tiko municipality include Moungo River, the Ombe River, Ndongo and Benyo streams which empty into the sea. The slow flow or stagnation of these streams favours the breeding of the snail intermediate host (Fig. 2a). Access to safe water is poor due to frequent non-flow of community and household water [25] and requirement for immediate payment of water fetched from private owned pipe-borne water sources (personal observation). Consequently, the local population makes frequent use of the streams for their daily household chores (Fig. 2b, c).

### Study design and sampling technique

**Fig. 3:**
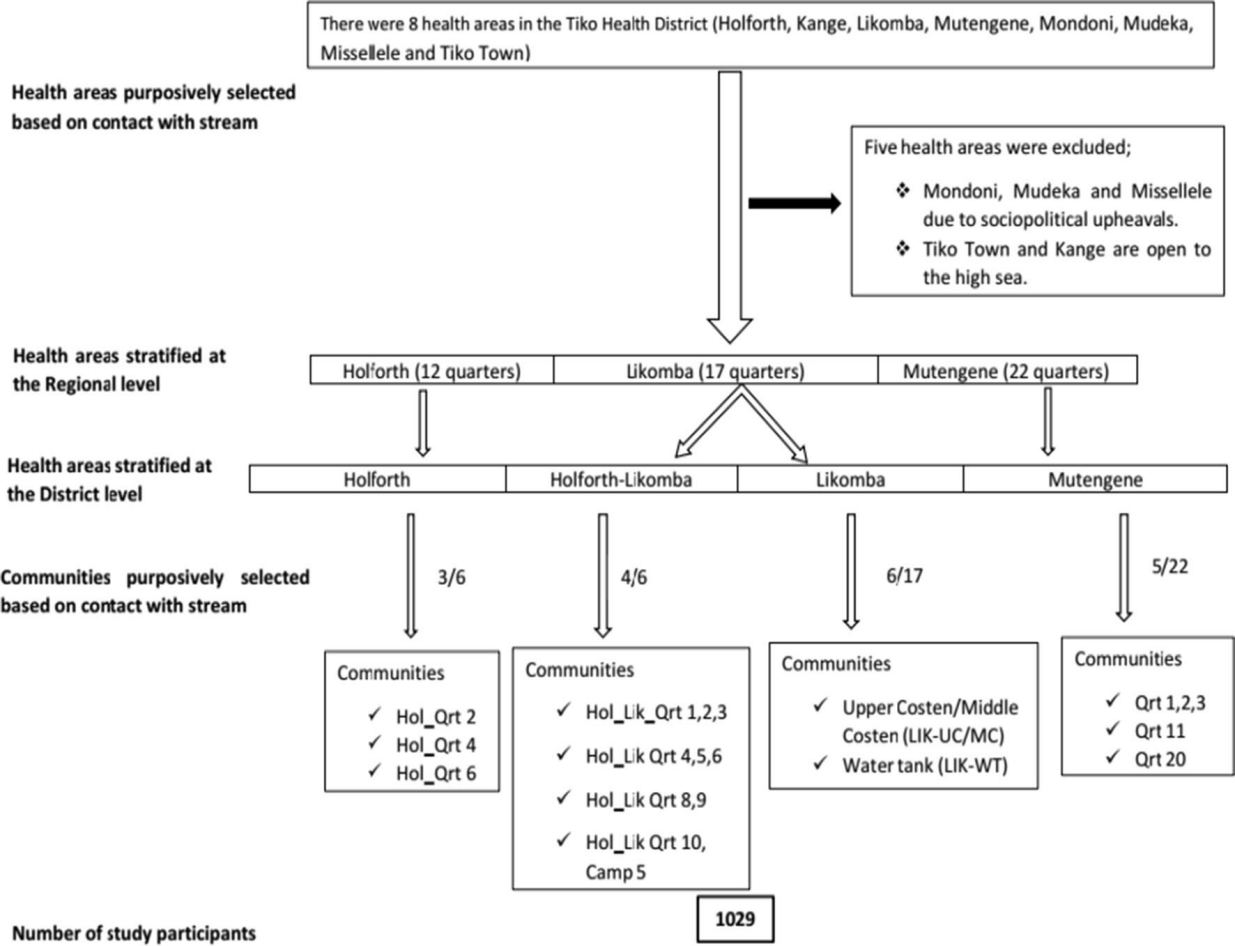
Flow diagram showing selection of study communities

The study was a cross-sectional community-based design carried out between June to August 2018. Out of the eight HAs, five were excluded from the study; Tiko town and Kange are open to the high sea while Mondoni, Mudeka and Missellele were the riskiest areas during the era of political unrest in the English-speaking part of the Country [26] (Fig. 3). Thus, three out of the eight HAs within the THD were selected for this study namely, Likomba, Holforth and Mutengene. However, at the district level, the Holforth HA (the largest and most populated) is divided into Holforth and Holforth-Likomba areas. In principle, four HAs were considered for the survey; Likomba, Holforth-Likomba, Holforth and Mutengene. Information on the population size of each selected HA and number of communities (neighbourhoods) were sorted from the Regional Delegation of Public Health, South West Region [27]. Twelve communities were purposively selected considering the water contact points [29] (Fig. 1) and a convenient sampling method was used to recruit study participants. The minimum sample size allocated per community was estimated from the total population size of the entire THD.

#### Sample size determination

The sample size for this study was determined based on the Yaro Yamane’s approach for finite population [30] using the formula; *n* = *N*/[1 + *N* (e)^2^].

where, *n* = the expected sample size, *N* = the finite population out of which the sample was drawn, e = the level of significance (or limit of tolerable error).

For this work, the estimated population size (*N*) from the 2017 population statistics was 124 500 [27], and the level of significance (e) was 0.05 or 5%. Thus, the estimated sample size = 124 500/[1 + 124 500 (0.05)^2^] = 124 500/312.25 ≈ 398. The minimum estimated sample size calculated per HA was 399. This was multiplied by 3 (i.e. number of HAs included in the survey) to give 1197. However, due to logistics, we had a sample size of 1029 consented participants which is well above 90% of the expected sample size calculated.

Ethical approval and administrative clearances were obtained prior to the commencement of the study. Community engagement in the selected communities were conducted with the support of the Health District, local chiefs, administrative and community health workers (CHW). Participants were invited to temporary data collection location in each community, and coordination was organized with the help of the CHW and leaders within the neighbourhood. Before enrolment into the study, informed consent/assent forms and information sheets (which highlights the purpose, risks and benefits of the study) were given to potential participants; for children less than 13 years old, assent was gotten from the parents or legal guardians, while for those less than 18 years old, consent was obtained from both the parent and the child. Individuals above 18 years gave their consent. The study team proceeded for sample collection upon obtaining consent/assent from the participants. A global positioning system (GPS) was used to get the accurate placement of the communities and water contact points.

### Inclusion and exclusion criteria

This study was designed to target individuals both males and females from five years and above living within the THD. Only respondents who have lived for at least two months in the study area and volunteered to participate in the study were enrolled. Enrolled individuals who failed to submit urine sample after the interview were excluded from the study.

### Questionnaire survey

Before data collection, a pilot testing of the questionnaire was done to ensure that the questions being asked, accurately reflect the information desired by the researcher and can be answered by the respondents. Challenges were identified and the changes effected accordingly. During the survey, participants were interviewed by a field researcher to record socio-demographic characteristics (age, gender, and residence), socioeconomic status (educational level and occupation) and history of *S.* *haematobium* infection (visible haematuria). Information on whether the participant had been living in the health district or came from other areas was noted. Access to the different water sources for household use (communal or household pipe-borne water), distance to open water sources as well as water-related activities such as agricultural practices, fishing, swimming, bathing, washing, laundry and crossing the stream were documented.

The degree of water contact was calculated using the formula, Σ (*R* × *F*), as described by Lima et al. [31]; where *R* is the score for the reason for the contact and *F* is the score for the frequency of contact. The reasons were given the following scores: 5 (for bathing, swimming, or playing in the streams), 4 (laundering or agricultural purpose), 3 (collecting water for the household, dish-washing or car/motor bike washing), and 2 (fishing or crossing the streams). The frequency of contacts was scored as 28 (daily or at least one contact a day), 4 (weekly or at least one contact a week), 2 (at least two contacts a month), and 1 (less than two a month). Totals of 2–99 were considered as degree I and ≥ 100 as degree II.

### Parasitology

#### Sample collection and processing

Each consented participant received a sterile, dry, screw-top, transparent, pre-labelled urine bottle. Participants were instructed to collect 20 ml of mid-stream urine sample in the container between 10:00 and 14:00. Immediately, samples were assessed for macrohaematuria and later tested for microhaematuria using urine reagent strips (URIT Medical Electronic Co., Ltd. P.R. China) following manufacturer’s instructions. Using the manufacturer colour chart, microhaematuria was detected as reagent strip colour reaction with varying levels indicated as trace, +, + +, + + +. The urine samples were later placed in a cool box and transported to the Malaria Research Laboratory, University of Buea, where, samples were processed using the syringe filtration technique [32].

Briefly, each urine specimen was well mixed, and 10 ml aliquot of urine sample was withdrawn into a syringe and filtered through a membrane filter (diameter, 25 mm; pore size, 8 µm) [Sterlitech Polycarbonate (PCTE) membrane filters, USA], fitted into a syringe holder, to separate the ova. Filter holder was then disassembled to expose the membrane. With the aid of a blunt forceps, each membrane was carefully removed and placed upside down onto a microscope slide. A drop of Lugol’s iodine was added and membranes examined for *S.* *haematobium* eggs under the × 10 objective of an Olympus NYUSA microscope (Wincom Company Ltd, China). Microhaematuria is considered as a proxy indicator of UGS and an accepted marker in the rapid diagnosis of *S.* *haematobium* infection in urine [33]. Thus, an individual was considered positive for *S.* *haematobium* when he/she was diagnosed positive by microscopic examination and/or urine reagent strip. The number of eggs was counted per 10 ml of urine and classified as light (< 50 eggs/10 ml of urine) or heavy (≥ 50 eggs/10 ml of urine) infection [34].

### Data management and statistical analysis

All data were entered, validated, and analysed using SPSS Statistics version 20 (SPSS Inc., Chicago, USA). Proportion of *S.* *haematobium* was compared between different groups (HAs, sex, age groups, level of education, occupation, source of water for household use and degree of contact) using Pearson Chi square test. Crude odds ratios were estimated, and factors associated with infection identified to be included in the multivariate logistic regression model. Variables that had a *P*-value < 0.20 in bivariate analysis or biological plausibility were included in the multivariate logistic regression model. Using the enter method, variables which showed independent association with infection at a significance level of *P* < 0.05 were retained in the model. Mean egg load was compared between groups using non-parametric Mann–Whitney and Kruskal Wallis test. Relationship between presence and intensity of *S.* *haematobium* eggs with microhaematuria and proteinuria was determined using Pearson Correlation. A *P*-value < 0.05 was considered significant.

### Cartographic analysis

ArcGIS Version 2.18 (Environmental Systems Research Institute, Inc., Redlands, USA) was used to evaluate the spatial distribution of UGS infection and behavioural risk factor (measured by degree of water contact). Types of improved water sources and distance to stream were mapped. Association between the distribution of UGS and explanatory variables were evaluated at community scale (community as geographic unit).

## Results

### Characteristics of study participants

A total of 1029 individuals (age range: 5–75 years) were enrolled from four HAs namely; Likomba, Holforth-Likomba, Holforth, and Mutengene HAs. For drinking water, domestic and recreational purposes, people living in THD use both the stream and improved water sources (pipe-borne water, boreholes, or protected wells). Despite high access (84.5%) to improved water facilities, more than half of the population reported the use of stream water (Table 1). A considerable number of people rely on the stream for two or more water-related activities such as water collection, laundry, bathing, playing, swimming, fishing, and farming. Compared with household pipe-borne water source, communal water supply is predominant (82.5%) in the area (Table 1), nevertheless, on most occasions, the water supply is irregular (personal observation).

**Table 1:** Characteristics of study participants

Characteristics	Number examined (*n*)	Proportion (%)
Health area		
Likomba	234	22.7
Holforth-Likomba	301	29.3
Holforth	243	23.6
Mutengene	251	24.4
Sex		
Male	525	51.0
Female	504	49.0
Age group (years)		
5–14	543	52.8
15–24	159	15.4
25–34	123	12.0
≥ 35	204	19.8
Level of education		
At least secondary	469	45.6
At least primary	560	54.4
Occupation		
Housewife	59	5.7
CDC worker	106	10.3
Pupil/student	659	64.0
Business/civil servant	121	11.8
Farmer	84	8.2
Stream usage		
Yes	563	54.7
No	466	45.3
Activities in the stream		
Water collection	46	4.5
Washing	96	9.3
Bathing	70	6.8
Playing/swimming	24	2.3
Farming/fishing	10	1.0
Two or more activities	317	30.8
Reported no contact	466	45.3
Frequency to stream		
Daily	284	50.4
Weekly	245	43.5
Monthly	34	6.1
Source of water		
Improved water source/stream^a^	563	54.7
Improved water source only	466	45.3
Pipe-borne water status		
Communal water	717	82.5
Household water	152	17.5

*CDC* Center for Disease Control and Prevention

^a^Communal/household pipe-borne water source, bore hold, protected well

### Prevalence of *S. haematobium* infection in the study area

*Schistosoma* *haematobium* infection was not detected in the Mutengene HA. Thus, the overall prevalence of *S.* *haematobium* infection in the Likomba, Holforth-Likomba and Holforth HAs was 31.5% [245/778, 95% confidence interval (*CI*): 28.3–34.8%]. The prevalence of microhaematuria was 24.9% (194/778).

**Fig. 4:**
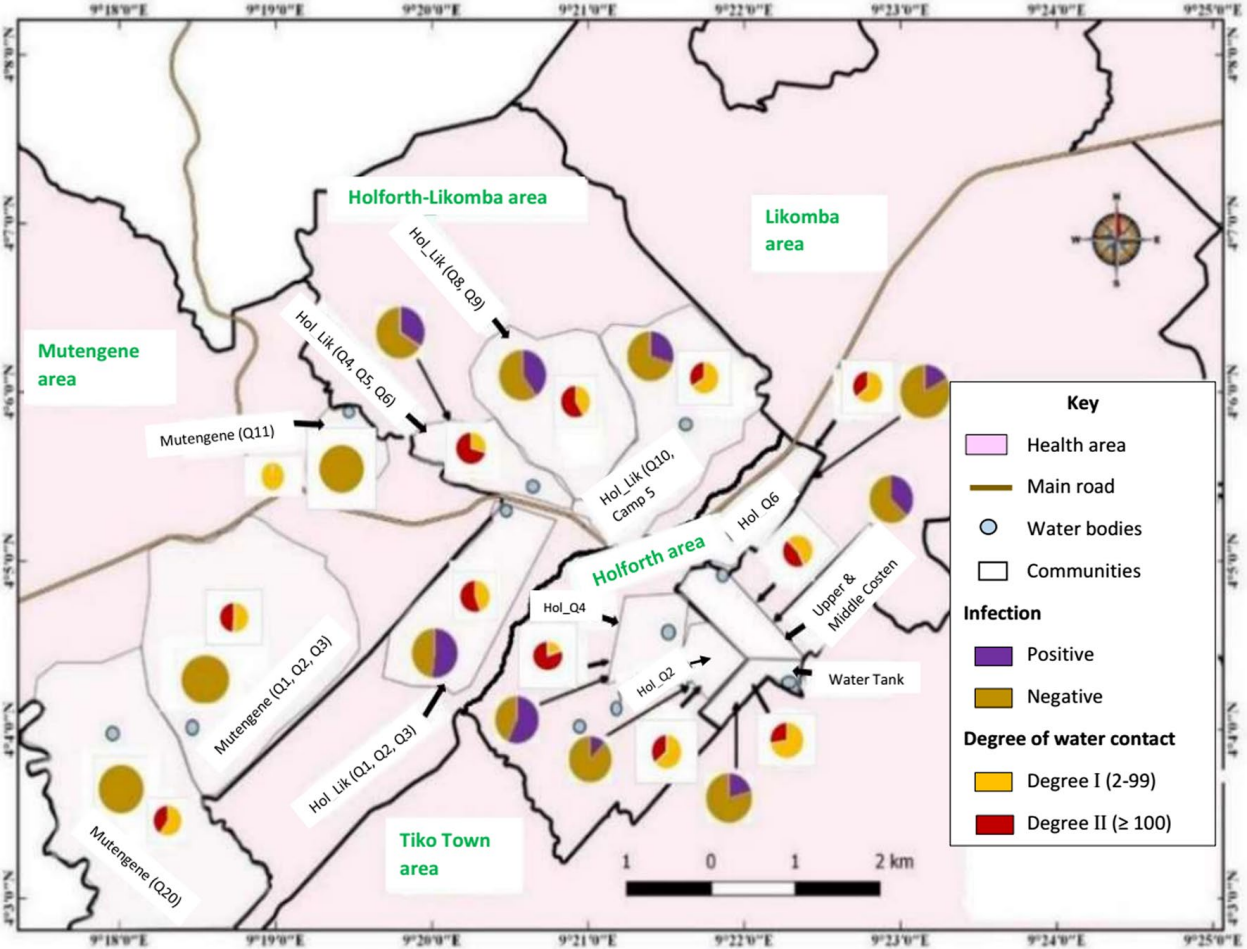
Prevalence of *Schistosoma* *haematobium* infection and degree of contact within different communities of Tiko Health District

Using urine filtration technique as gold standard, the sensitivity and specificity of urine reagent strip in the diagnosis of *S.* *haematobium* infection were 70.3% (95% *CI*: 63.1–76.7%) and 87.9% (95% *CI*: 85.1–90.3%), respectively. The Holforth-Likomba HA had the highest prevalence at 38.9% and Holforth HA having the least prevalence (23.5%). Figure 4 shows the spatial distribution of the twelve communities sampled in the four HAs and the respective prevalence of *S.* *haematobium* infection ranging from 0 to 56.9%. Nine out of twelve communities were affected. The prevalence of infection varied significantly (*χ*^2^ = 66.21; *P* < 0.001) among the affected communities. According to the endemicity of the disease, UGS was hyperendemic (prevalence ≥ 50%) in Holforth quarter 4 (HOL Q4) and Holforth-Likomba quarter 1, 2, 3 (HOL-LIK Q1, 2, 3) neighbourhoods, and mesoendemic (10% ≤ prevalence < 50%) in the other five communities (Additional file 1).

**Fig. 5:**
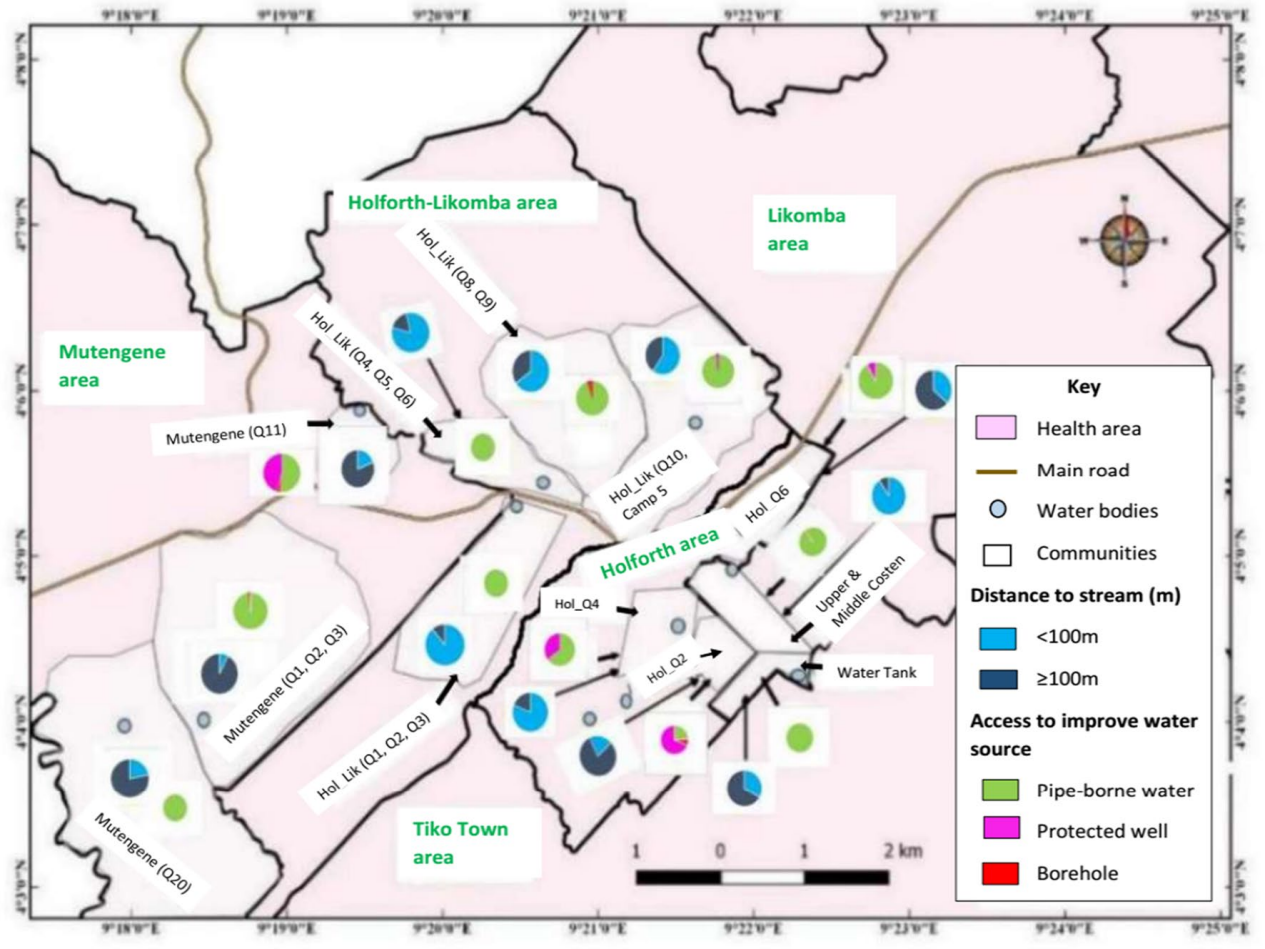
Distribution of proximity to stream and access to improved water source within different communities of Tiko Health District

At the community level, the prevalence of UGS had a significant positive linear relationship (*r* = 0.8, *P* = 0.01) with the intensity of contact with water body; as the degree of contact with water increases, the chances of its inhabitants having *S.* *haematobium* infection increased. Most of the individuals living in highly affected communities (HOL Q4 and HOL-LIK Q1, 2, 3) indicated residence in proximity (< 100 m) to water bodies (Fig. 4). In addition, the distance of residence to stream was a determinant of infection with *S.* *haematobium* (*χ*^2^ = 6.65; *P* = 0.010). This association was particularly evident for HOL Q2 (*χ*^2^ = 9.00; *P* = 0.003) (Additional file 2). More improved water sources (*χ*^2^ = 433.65; *P* < 0.001) were noted in the Holforth HA (Additional file 3). It is worth noting that, HOL Q2 and HOL Q6 communities were less affected by UGS. Individuals living in these neighbourhoods either live far (≥ 100 m) from surface water points or have access to more improved water sources as well as make less contact with surface water (Figs. 4 and 5).

The univariate analysis of determinant factors of *S.* *haematobium* in the THD based on the surveyed sociodemographic and water contact behaviour is summarised in Table 2. The infection was frequent among the age group 5–14 years, pupils/students and individuals who had attained at least secondary level of education. Also, a higher prevalence was observed among those, who made daily contact with stream (*χ*^2^ = 173.6; *P* < 0.001), reported playing and swimming in the stream as well as carry out multiple water contact activities (*χ*^2^ = 117.3; *P* < 0.001) when compared with others in their respective categories.

**Table 2:** Univariate analysis of socio-demographic and water contact behaviour associated with urogenital schistosomiasis in the study population

Variable	Category	*Schistosoma* *haematobium* infection, % (*n*)	*χ*^2^	*P* value
Health area	Likomba	30.3 (71)	15.0	< 0.05
	Holforth_Likomba	38.9 (117)		
	Holforth	23.5 (57)		
Sex	Male	32.9 (138)	0.88	0.35
	Female	29.8 (107)		
Age group (years)	5–14	37.7 (156)	22.0	< 0.05
	15–24	31.7 (39)		
	25–34	25.8 (24)		
	≥ 35	17.6 (26)		
Level of education	Secondary	35.4 (126)	4.6	0.03
	Primary	28.2 (119)		
Occupation	Housewife	30.0 (12)		
	CDC worker	18.5 (17)	18.5	< 0.05
	Student/Pupil	36.5 (184)		
	Business/Civil servant	26.3 (21)		
	Farmer	17.7 (11)		
Stream activity	Two/more activities	53.5 (137)	117.3	< 0.05
	Water collection	33.3 (14)		
	Washing	40.3 (25)		
	Bathing	28.3 (15)		
	Playing/swimming	53.8 (7)		
	Farming/fishing	25.0 (2)		
	No activity	13.1 (45)		
Stream frequency	Daily	62.4 (159)	173.6	< 0.05
	Weekly	21.5 (32)		
	Monthly	30.0 (9)		
Degree of contact^a^	Degree II (≥ 100)	64.2 (145)	62.0	< 0.05
	Degree I (2–99)	26.4 (55)		

*CDC* Center for disease control and prevention

^a^Σ (*R* × *F*), where *R* is the score for the reason for the contact and *F* the score for the frequency of contact

### Intensity of *S. haematobium* egg excretion and associated clinical outcome

Egg excretion was recorded for 172 among whom 61.4% (105/172) (< 50 eggs/ 10 ml urine) had light infection while 38.6% (66/172) (≥ 50 eggs/10 ml of urine) had heavy infection. The geometric mean (GM) egg count was 28.7 eggs per 10 ml of urine (range: 2–450) with no significant variability (*P* = 0.55) among the different HAs (Table 3). Mean egg count decreased significantly (*P* = 0.02) with increased age, where the 5–14 years age group had the highest egg load excretion and the least egg counts were recorded among older individuals (25–34 and ≥ 35 years). In addition, intense contact with stream (degree II) was associated with higher egg load excretion than less contact with stream (degree I). The difference was highly significant (*P* < 0.001). While more individuals with light infection (*P* < 0.001) showed no urothelial clinical symptoms when compared with heavy infection, higher mean egg count was associated with the presence of microhaematuria and proteinuria (Table 3).

**Table 3:** Intensity of *Schistosoma* *haematobium* infection in the study population stratified according to socio-demographic, water contact behaviour and clinical presentation

Factors	Intensity of infection		*χ*^2^	*P* value^$^	GM egg load/10 ml urine	Range/10 ml urine	*P* value
	Light % (*n*)	Heavy % (*n*)					
Health area							
Likomba	60.4 (29)	39.6 (19)	0.46	0.79	25.8	2–317	0.55^b^
Holforth_Likomba	60.0 (48)	40.0 (32)			26.9	2–450	
Holforth	65.9 (29)	34.1 (15)			36.4	2–225	
Sex							
Male	59.3 (67)	40.7 (46)	0.76	0.38	29.5	2–450	0.76^a^
Female	66.1 (39)	33.9 (20)			27.3	2–400	
Age group (years)							
5–14	57.6 (72)	42.4 (53)	5.57	0.13	34.4	2–450	0.02*^b^
15–24	69.0 (20)	31.0 (9)			22.2	2–330	
25–34	90.9 (10)	9.1 (1)			10.2	2–53	
≥ 35	57.1 (4)	42.9 (3)			16.8	2–216	
Level of education							
Secondary	57.0 (49)	43.0 (37)	1.53	0.21	29.4	2–317	0.65^a^
Primary	66.3 (57)	33.7 (29)			28.1	2–450	
Occupation							
Housewife	80.0 (4)	20.0 (1)	3.81	0.34	25.8	9–57	0.17^b^
CDC worker	81.8 (9)	18.2 (2)			11.6	2–216	
Student/Pupil	59.4 (85)	40.6 (58)			31.9	2–450	
Business/Civil servant	75.0 (6)	25.0 (2)			17.8	3–330	
Farmer	40.0 (2)	60.0 (3)			24.7	2–183	
Stream activity							
Two/more activities	58.6 (68)	41.4 (48)	11.4	0.08	31.3	2–400	0.04*^b^
Water collection	75.0 (6)	25.0 (2)			17.1	2–450	
Washing	81.8 (18)	18.2 (4)			25.3	5–330	
Bathing	53.3 (8)	46.7 (7)			31.7	3–280	
Playing/swimming	33.3 (2)	66.7 (4)			55.4	7–215	
Farming/fishing	0.0 (0)	100.0 (1)			54.0	–	
No contact	100.0 (4)	0.0 (0)			2.9	2–4	
Stream frequency							
Daily	55.2 (80)	44.8 (65)	16.37	0.001	33.8	2–450	< 0.001*^b^
Weekly	94.1 (16)	5.9 (1)			15.6	5–51	
Monthly	100.0 (6)	0.0 (0)			14.5	7–34	
No contact	100.0 (4)	0.0 (0)			2.9	2–4	
Degree of contact							
Degree II (≥ 100)	53.7 (72)	46.3 (62)	13.54	< 0.001	35.9	2–400	< 0.001*^a^
Degree I (2–99)	88.2 (30)	11.8 (4)			15.5	2–450	
Microhaematuria							
Yes	50.4 (61)	49.6 (60)	21.7	< 0.001	40.1	2–450	< 0.001*^a^
No	88.2 (45)	11.8 (6)			13.0	2–280	
Dysuria							
Yes	85.7 (12)	14.3 (2)	3.74	0.05	22.8	2–450	0.47 ^a^
No	59.5 (94)	40.5 (64)			29.3	2–400	
Frequent urination							
Yes	37.5 (3)	62.5 (5)	2.06	0.15	22.8	2–450	0.25^a^
No	62.8 (103)	37.2 (61)			29.3	2–400	
Proteinuria							
Yes	48.3 (43)	51.7 (46)	13.82	< 0.001	41.2	2–450	< 0.001*^a^
No	75.9 (63)	24.1 (20)			19.5	2–280	

*GM* geometrical mean, *CDC* Center for Disease Control and Prevention

^a^Difference in egg load determined by Mann–Whitney *U* test

^b^Difference in egg load determined by Kruskal–Wallis test

^**$**^Values are from Pearson Chi square test

*Statistically significant *P* value

### Risk factors associated with *S. haematobium* infection in the THD

The binary logistic regression analysis presents determinant factors associated with the risk of *S.* *haematobium* infection based on sociodemographic factors and water contact behaviour in THD (Table 4). The most important factors associated with infection in the study area were age, secondary level of education and degree of water contact. Individuals in the younger age group (5–14 years) were observed to be 3.7 times (95% *CI*: 1.1–12.2) more likely to be infected when compared with those of the older age groups. More so, individuals who had secondary level of education were twice (95% *CI*: 1.2–3.2) more likely to be infected with *S.* *haematobium* compared with those who had at most primary level of education. Participants who were classified as having high degree contact (degree II) with water had 5.2 times increased odds (95%* CI*: 3.4–8.1) of infection.

**Table 4:** Risk factors associated with *Schistosoma* *haematobium* infection in the Tiko health district

Factors	% (*n*) positive	Unadjusted *OR*	95% *CI*	*P* value^$^	^#^Adjusted *OR*	95% *CI*	*P* value
Health area							
Likomba	30.3 (71)	1.4	0.9–2.1	< 0.05	1.3	0.7–2.3	0.44
Holforth_Likomba	38.9 (117)	2.1	1.4–3.0		1.2	0.7–2.1	0.40
Holforth	23.5 (57)	REF			REF		
Sex							
Male	32.9 (138)	1.07	0.9–1.2	0.35	1.2	0.8–1.9	0.33
Female	29.8 (107)	REF			REF		
Age group (years)							
5–14	37.7 (156)	3.8	1.8–4.5	< 0.05	3.7	1.1–12.2	0.03
15–24	31.7 (39)	2.18	1.2–3.8		2.5	0.8–8.2	0.13
25–34	25.8 (24)	1.63	0.9–3.1		2.6	0.9–7.3	0.07
≥ 35	17.6 (26)	REF			REF		
Level of education							
Secondary	35.4 (126)	1.2	1.0–1.4	0.03	2.0	1.2–3.2	0.005
Primary	28.2 (119)	REF			REF		
Occupation							
Housewife	30.0 (12)	2.0	0.8–5.1	< 0.05	1.2	0.3–6.2	0.78
CDC worker	18.5 (17)	1.0	0.4–2.4		0.9	0.2–3.1	0.83
Pupil/student	36.5 (184)	2.7	1.3–5.2		1.0	0.3–3.7	0.94
Business/Civil servant	26.2 (21)	1.6	0.7–3.7		2.5	0.7–8.4	0.15
Farmer	17.7 (11)	REF			REF		
Source of pipe-borne water							
Household pipe-borne water	34.7 (195)	1.0	0.9–1.1	0.32	NA		NA
Communal pipe-borne water	29.8 (34)	REF					
Degree of water contact							
Degree II (≥ 100)	64.2 (145)	2.0	1.7–2.5	< 0.05	5.2	(3.4–8.1)	< 0.05
Degree I (2–99)	26.4 (55)	REF			REF		

*CDC* Center for Disease Control and Prevention, *CI* confidence interval, *OR* odd ratio, *NA* not applicable

^$^Pearson Chi-square test

^#^Adjusted *OR* using multivariate regression analysis

## Discussion

The current study reports on the distribution of *S.* *haematobium* infection in THD and an unmapped transmission focus in the Mount Cameroon area [24]. Our study confirms that *S.* *haematobium* transmission occurs in Likomba, Holforth-Likomba and Holforth HAs with the occurrence of infection at 31.5%. No evidence of human host infection was recorded in Mutengene HA. Although, THD can be classified as moderate risk endemic area [11], the infection levels in affected communities varied from meso to hyperendemic (12.0–56.9%). The occurrence of UGS in THD is linked partly to the presence and use of many fresh waterbodies (such as Moungo River) and its tributaries, which intersperse the Mount Cameroon Area. Also, proximity to stream, intense water contact activities and inadequate improved water sources are important drivers of transmission of *S.* *haematobium* in the district.

Compared with the prevalence of UGS in THD, lower rates have been observed in some urban and semi-urban settings in other Regions of Cameroon: 1.7% in Kékem [35] and 22.9% in Maroua [36]. These areas are targeted regularly for control of schistosomiasis and geohelminths and thus account for the lower prevalence of schistosomiasis in these areas [35]. It is interesting that Mutengene HA which is about 250 m away from Likomba HA had zero prevalence. Mutengene is located at a higher altitude [220 m above sea level (asl)] with a hilly topographic terrain characterized by fast flow of the “Ndongo” stream which slows as it meanders across the Tiko and Limbe towns. The influence of topography on *Schistosoma* parasite transmission has been emphasized [37]. A study in Nigeria reported absence of schistosomiasis in communities located at higher altitudes [38]. Conversely, lower altitudes could have a significant impact on the *Schistosoma* snail vector distributions, which are likely to be more concentrated in areas where there is a slow water current [39]. This may explain the predominance of UGS in the Likomba and Holforth HAs located at a lower altitude (18–80 m asl). It is also possible that far distance to and infrequent contact with water bodies may prevent establishment and transmission of *S.* *haematobium* in the Mutengene HA, however, this remains to be determined.

Among the nine affected communities, five namely: LK-UC/MC; HOL-LKQ1,2,3; HOL-LKQ4,5,6; HOL-LKQ 8,9 and HOLQ6 had infection > 31.0% with HOL-LKQ 1,2,3 (52.0%) and HOL-LKQ4 (56.9%) observed as high-risk communities (≥ 50%). Majority (> 80%) of the residents in these communities live very close to the stream and are equally involved in intense contact with surface water. On the other hand, low prevalence of infection in communities like HOL Q2 (12.5%) and HOL Q6 (17.0%) may be linked to the fact that most of the people live far (≥ 100 m) (80.0% for HOL Q2 and 63.8% for HOL Q6) from open water surface and have access to alternative improved water sources such as protected wells and boreholes [40]. Our findings strongly demonstrate the focal transmission pattern of schistosomiasis which suggests strategic control of this disease. The maps obtained provide information about the areas where studies and control efforts need to be focused. Preventive chemotherapy with praziquantel should be immediately put in place to prevent morbidity and interrupt transmission. Nevertheless, a malacological study on the distribution of snail intermediate hosts is crucial to elucidate the epidemiology of infection in the THD.

The prevalence of *S.* *haematobium* decreased with age where school-aged children (5–14 years) are associated with higher odds and intensity of *S.* *haematobium* infection. This agrees with trends established in surveys carried out in Cameroon [21, 41] and other parts of Africa [18, 42]. Individuals of this age group are predisposed to schistosome infections due to their active life; hence increased water contact activities with cercaria infested streams [18]. Children are the most infected group of people in endemic areas, thus contributes significantly to the potential contamination of the aquatic environment [43]. Age-acquired immunity to reinfection underlies the declining trend in infection with increasing age [44].

The education level of the inhabitants was also strongly associated with the transmission of *S.* *haematobium*. Contrary to findings in Munyenge, Mount Cameroon Area [45], secondary level of education increased the odds of infection when compared with primary level of education. There is no distinct reason why individuals with secondary education had higher prevalence of infection. However, from statistical analysis, it is reasonable to suggest that the active age group (11–24 years) may fall more into the secondary education category. In Nigeria, the age group 11–20 years was the most active age group frequently engaging in activities that bring them in contact with infested water bodies [46].

The sex dependent pattern of schistosome infections is widely reported [43]. Conversely, the prevalence of infection in males and females was similar in our study. It is likely that, socioeconomic conditions, and habits in the THD could modify sex-biased tendencies towards water contact behaviour [18, 40]. Males and females equally participate in water activities such as bathing, swimming, and laundry, which served as major predisposing factors. Previous studies in Nigeria [47], Cote d’Ivoire [18] and Cameroon [8] have reported similar observations.

Despite the presence of improved water facilities in the HAs, about 50% of the population visit the stream daily and one-third of those who use the stream, engage in two or more water-related activities. In most countries where schistosomiasis is endemic, inadequate access to clean water sources is major concern [48]. Distance from home, limited number of communal piped-borne water sources and requirement for immediate payment of piped-borne water are leading causes for limited access to water [45, 49]. Many natural water bodies, infested with snails and schistosome cercariae, are common sources for domestic water in most schistosomiasis endemic areas [50]. In THD, the flow of pipe-borne water is inconsistent, consequently, streams and springs are common sources of water for majority of the people in this District. Usually people become infected with schistosomes when they make contact with infested water and cercariae penetrate the skin. Humans excrete eggs in water bodies through faecal or urine contamination, the eggs hatch into miracidia which finds and penetrate the snail intermediate hosts. In the snail host, the parasite develops into cercariae which are infective to humans [51]. The high dependence of the population on natural water bodies avails each water contact activity a potential risk factor of *S.* *haematobium* infection [52]. Intense water contact activity and daily visitation to stream (degree II) was associated with increased risk and intensity of *S.* *haematobium* infection. This confirms previous reports in other endemic foci in Cameroon [8, 45, 53] and elsewhere [54]. Water contact at any point in time is linked mostly to practices including domestic activities and bathing. Laundry, bathing, and recreational swimming are the activities that cause the most exposure to cercaria-infested water because these activities do involve the immersion of large body parts, for long periods [31].

This study had some limitations. Only four out of eight HAs were surveyed due to inaccessibility. Future studies to survey the other communities are important and will provide a clearer picture of UGS in the entire district. Secondly, this study relied only on statistical methods to establish the transmission of *S.* *haematobium* infection and associated factors in THD. Malacological studies are crucial to confirm that the streams are infested with snails which harbour the infective larvae of the parasite.

## Conclusions

THD is a moderate risk endemic focus for UGS with an overall prevalence of 31.5%. Transmission occurs in Likomba, Holforth-Likomba and Holforth HAs and was not observed in Mutengene HA. The infection level varied significantly among affected communities from meso- to hyperendemic (12.0–56.9%). Proximity to stream, intense contact with stream and access to more improved water sources determined infection status of the communities. Among the participants, determinant factors associated with risk of *S.* *haematobium* infection and higher egg load excretion were younger age (5–14 years) and high degree of contact with open water surface. A malacological study on snail intermediate host (s) distribution and infectivity is fundamental to elucidate the mechanisms of *S.* *haematobium* transmission in THD. Nevertheless, control measures to effectively control and eventually eliminate the infection in this area will entail mass drug administration of praziquantel to affected communities, provision of safe and adequate water supply, health education, and snail control.

## Supplementary Information

**Additional file 1:** Variation in the occurrence of *Schistosoma haematobium* infection among affected communities in Tiko Health District

**Additional file 2:** The prevalence of *Schistosoma haematobium* infection in relation to distance to stream in the different communities in Tiko Health District

**Additional file 3:** Reported access to improved water sources among the affected communities in Tiko Health District

## Data Availability

All data sets on which the conclusions of the research rely are presented in the paper and its supplementary file. However, data is available from the corresponding author upon reasonable request.

## Abbreviations

THD: Tiko Health District
CHW: Community Health Worker
UGS: Urogenital schistosomiasis
SWR: South west region
WASH: Water, sanitation and hygiene
