# Supplementary material for: Distribution and factors associated with urogenital schistosomiasis in the Tiko Health District, a semi-urban setting, South West Region, Cameroon
Source: Infect Dis Poverty. 2021 Apr 12;10:49. doi: 10.1186/s40249-021-00827-2 (PMC8042887; doi:10.1186/s40249-021-00827-2)
Supplement: Supplementary file 1 — Additional file 1: Variation in the occurrence of Schistosoma haematobium infection among affected communities in Tiko Health District [file 40249_2021_827_MOESM1_ESM.docx]

**Additional file 1: variation in the occurrence of *S. haematobium* infection among affected communities in THD**

| **Communities** | **N** | ***S. haematobium* infection, %(n)** |
| --- | --- | --- |
| LIK-UC/MC | 137 | 37.2(51) |
| LIK-WT | 97 | 20.6(20) |
| HOL-LIK Q1,2,3 | 75 | 52.0(39) |
| HOL-LIK Q4,5,6 | 62 | 33.9(21) |
| HOL-LIK Q8,9 | 80 | 40.0(32) |
| HOL-LIK Q10, Camp 5 | 84 | 29.8(25) |
| HOL Q2 | 92 | 12.0(11) |
| HOL Q4 | 51 | 56.9(29) |
| HOL Q6 | 100 | 17.0(17) |
| **Level of significance** | **χ2 = 66.21; p < 0.001** | |
