# Supplementary material for: Distribution and factors associated with urogenital schistosomiasis in the Tiko Health District, a semi-urban setting, South West Region, Cameroon
Source: Infect Dis Poverty. 2021 Apr 12;10:49. doi: 10.1186/s40249-021-00827-2 (PMC8042887; doi:10.1186/s40249-021-00827-2)
Supplement: Supplementary file 2 — Additional file 2: The prevalence of Schistosoma haematobium infection in relation to distance to stream in the different communities in Tiko Health District [file 40249_2021_827_MOESM2_ESM.docx]

**Additional file 2: The prevalence of *S. haematobium* infection in relation to distance to stream in the different communities in THD**

| **Communities** | **Distance to stream**  **% (n)** | | **Prevalence of *S. haematobium* infection %(n)** | |
| --- | --- | --- | --- | --- |
|  | **<100 m** | **≥100 m** | **<100 m** | **≥100 m** |
| LIK-UC/MC | 90.5(76) | 9.5(8) | 50.0(38) | 37.5(3) |
| LIK-WT | 33.3(12) | 66.7(24) | 33.3(12) | 33.3(8) |
| HOL-LIK Q1,2,3 | 88.9(48) | 11.1(6) | 50.0(24) | 83.3(5) |
| HOL-LIK Q4,5,6 | 82.8(48) | 17.2(10) | 31.3(15) | 40.0(4) |
| HOL-LIK Q8,9 | 65.2(30) | 34.8(16) | 66.7(20) | 62.5(10) |
| HOL-LIK Q10, Camp 5 | 59.6(28) | 40.4(19) | 50.0(14) | 36.8(7) |
| HOL Q2 | 20.0(5) | 80.0(20) | 60.0(3) | 5.0(1) |
| HOL Q4 | 81.1(30) | 18.9(7) | 70.0(21) | 85.7(6) |
| HOL Q6 | 36.2(17) | 63.8(30) | 52.9(9) | 26.7(8) |
| **Level of significance** | **χ^2^ = 1081.56; p < 0.001** | | **χ^2^ = 6.65; p = 0.010** | |
