# Supplementary material for: Distribution and factors associated with urogenital schistosomiasis in the Tiko Health District, a semi-urban setting, South West Region, Cameroon
Source: Infect Dis Poverty. 2021 Apr 12;10:49. doi: 10.1186/s40249-021-00827-2 (PMC8042887; doi:10.1186/s40249-021-00827-2)
Supplement: Supplementary file 3 — Additional file 3: Reported access to improved water sources among the affected communities in Tiko Health District [file 40249_2021_827_MOESM3_ESM.docx]

**Additional file 3: Reported access to improved water sources among the affected communities in THD**

| **Communities** | **Pipe-borne water %(n)** | **Borehole %(n)** | **Protected well %(n)** |
| --- | --- | --- | --- |
| LIK-UC/MC | 99.3(136) | 0.7(1) | 0(0) |
| LIK-WT | 100.0(97) | 0(0) | 0(0) |
| HOL-LIK Q1,2,3 | 100.0(75) | 0(0) | 0(0) |
| HOL-LIK Q4,5,6 | 100.0(62) | 0(0) | 0(0) |
| HOL-LIK Q8,9 | 95.0(76) | 5.0(4) | 0(0) |
| HOL-LIK Q10, Camp 5 | 98.8(83) | 0(0) | 1.2(1) |
| HOL Q2 | 23.9(22) | 7.6(7) | 68.5(63) |
| HOL Q4 | 64.7(33) | 0(0) | 35.3(18) |
| HOL Q6 | 92.0(92) | 0(0) | 8.0(8) |
| **Level of significance** | **χ^2^ = 433.65; p < 0.001** | | |
